# A Text-Book of the Theory on Practice of Medicine

**Published:** 1893-08

**Authors:** 


A TEXT-BOOK ON THE THEORY AND PRACTICE OF MEDICINE BY AMERICAN TEACHERS. Edited by William Pepper, M. D., LL. D., Provost and Professor of the Theory and Practice of Medicine and Clinical Medicine in the University of Pennsylvania. In two royal octavo volumes, of about 1000 pages each. For sale by subscription only. Price, per volume, cloth, $5; sheep, $6; half Russia, $7. W. B. Saunders, publisher, 913 Walnut Street, Philadelphia, Pa.

Volume I of this magnificent work is now before us, and after reading it over carefully we do not hesitate to say that it is one of the best, if not *the* best work on the theory and practice of medicine in the English language.

When the enterprising publisher, W. B. Saunders, announced some months since, the early appearance of this work, to be edited by Dr. Pepper, with the assistance of a number of America's greatest teachers, the profession at large naturally expected a work without a superior, and in this expectation they will not be disappointed. Each author has selected the subject in which he is most interested, and to which he has devoted the most study, time and attention; and all of the authors being teachers, they know how to present these subjects to their readers in a form the most impressive and most easy of comprehension. Each article is an exhaustive treatise, giving the latest facts as regards the causation, symptomatology, diagnosis, prognosis and treatment. The opening chapter is by Dr. John S. Billings, on the subject of hygiene. No one in this country is better qualified than Dr. Billings to give instructions on this subject; and under the sub-headings of Predisposing Causes of Disease, Mental Causes of Disease, Micro-organisms, Immunity, Disinfection, Isolation, Food, Exercise, Clothing and Bedding, Occupation, Habitation, Water Supply, Sewage Disposal, House Sewerage, Ventilation, Disposal of the Dead and Sanitary Jurisprudence, the principal facts connected with the subject are brought out and fully elucidated. Dr. Pepper furnishes an article on each of the following subjects: Ephemeral fever and simple continued fever, typhoid fever, typhus fever, relapsing fever, cerebro-spinal fever, influenza, dengue, milliary fever, milk sickness, mountain fever, septicæmia and pyaemia, and leprosy.

Dr. James T. Whittaker writes on the subjects of scarlatina, measles, rubeola, small-pox, mumps, whooping-cough, tetanus, actinomscosis, anthrax, hydrophobia, trichinosis, glanders, and foot-and-mouth disease.

By Dr. W. Gilman Thompson: Acute miliary tuberculosis, scrofula, syphilis, diphtheria, erysipelas, malarial fevers, cholera, and yellow fever.

By Dr. H. C. Wood: General symptomatology of diseases of the nervous system, mental diseases, functional nervous diseases, syphilis of the nervous system, organic diseases of the spinal cord and its membranes.

By Dr. Wm. Osler: Organic diseases of the brain, diseases of the nerves, diseases of the muscles, vaso-motor and trophic disorders.

To undertake to compliment any one of these articles, would be doing an injustice to all the rest. They are all deserving of the highest praise and commendation.

The mechanical execution of the book is excellent, and it contains numerous wood cuts and colored plate illustrations to elucidate the text whenever necessary. We predict a large sale for the work, as no progressive physician can afford to be without it.

